# The relationship between lateral femoral condyle ratio measured by MRI and anterior cruciate ligament injury

**DOI:** 10.3389/fbioe.2024.1362110

**Published:** 2024-03-27

**Authors:** Yang Sun, Yun Tang

**Affiliations:** ^1^ Department of Sports Medicine, The First People’s Hospital of Lianyungang, Lianyungang, China; ^2^ Clinical Research Center, The First People’s Hospital of Lianyungang, Lianyungang, China

**Keywords:** anterior cruciate ligament, ACL, lateral femoral condyle ratio, LFCR, MRI, risk factor

## Abstract

**Background:**

Previous studies have shown that the lateral femoral condyle ratio (LFCR) measured by X-ray has a significant relationship with the anterior cruciate ligament (ACL) injury. However, few relevant studies have been performed on LFCR measured by magnetic resonance imaging (MRI).

**Purpose:**

(1) To evaluate the relationship between LFCR measured by MRI and ACL injury or rerupture. (2) To compare the LFCR measured by MRI with existing bony morphological risk factors and screen out the most predictive risk factors for primary ACL injury or rerupture.

**Study Design:**

Cohort study; Level of evidence, 3.

**Methods:**

Totally 147 patients who underwent knee arthroscopic surgery from 2015 to 2019 with minimum follow-up of 48 months were retrospectively evaluated. Patients were placed into three groups: 1) the control group of patients with simple meniscus tears without ligament injury; 2) the primary noncontact ACL injury group; 3) ACL rerupture group (ACL reconstruction failure). The LFCR measured by MRI and other previous known risk factors associated with MRI (notch width index, medial tibial slope, lateral tibial slope, medial tibial depth, lateral tibial height) were performed to evaluate their predictive value for ACL injury and rerupture. All the risk factors with *p* < 0.01 according to univariate analysis were included in the logistic regression models. Receiver operating characteristic (ROC) curves were analyzed for sensitivity, specificity, cut-off, and area under the curve (AUC). Z tests were used to compare the AUC values.

**Results:**

The LFCR measured by MRI was obviously higher in primary ACL injury group (0.628 ± 0.020) and in ACL rerupture group (0.625 ± 0.021) than that in the control group (0.593 ± 0.030). The best risk factor was the LFCR with a cut-off of 0.602 (AUC, 0.818; 95% CI, 0.748–0.878; sensitivity, 90%; specificity, 66%). When combined with lateral tibial slope (cutoff, 7°) and lateral tibial height (cutoff, 3.6 mm), the diagnostic performance was improved significantly (AUC, 0.896; 95% CI, 0.890–0.950; sensitivity, 87%; specificity, 80%).

**Conclusion:**

The increased LFCR measured by MRI was associated with a significantly higher risk for ACL injury or rerupture. The combination of LFCR, lateral tibial slope and lateral tibial height were the most predictive risk factors. This may help clinicians identify susceptible individuals and allow precision approaches for better prevention, treatment and management of this disease.

## Introduction

The incidence of anterior cruciate ligament (ACL) injury among athletes and adolescents is increasing year by year (Beck et al., 2017). The profound effects of ACL injury on quality of life have drawn increased attention. Anterior cruciate ligament reconstruction is the gold standard of treatment for ACL injury (Attia et al., 2014), but the high re-rupture rate has plagued clinicians throughout the world (Lim et al., 2019). Among the leading research topics in sports and orthopedics is ACL injury risk factors (Carcia et al., 2019). There are a number of factors that may play a role in the risk of ACL injury, including gender (Herzog et al., 2017; Webster and Feller, 2016), body mass index (BMI) (DiSilvestro et al., 2019; Kaarre et al., 2023), neuromuscular factors (Smith et al., 2012), hormone (Smith et al., 2012), bony morphological factors (Bayer et al., 2020). Within this scope, we focused on the influence of bony morphological factors on ACL injury.

Multiple bony morphological factors have been reported to be related to the risk of ACL injury, such as the tibial slope (Hashemi et al., 2010; Hohmann et al., 2021), the medial tibial depth (MTD) (Hashemi et al., 2010), the lateral tibial height (LTH) (Kujala et al., 1992), the narrow intercondylar notch (NWI) (Hao et al., 2023). The relationship between the morphological characteristics of the distal femoral condyle and the incidence of noncontact ACL injury has become the hotspot of current research. In particular, the sphericity of lateral femoral condyle is considered to have intimate associations with the rotation and Pivot-shift of knee. Based on the lateral femoral condyle index (LFCI), Hodel et al. (2019) demonstrated that the decreased LFCI was associated with primary ACL injury. Nevertheless, the reproducibility and reliability of this measurement have been questioned by recent studies (Li et al., 2020; Nowak et al., 2022).

Comparably, in order to establish a specific standardized computational method to quantify the morphological characteristics of lateral femoral condyle, Pfeiffer et al. (2018) consider that the lateral femoral condyle ratio (LFCR) measured by X-ray is associated with a higher risk of noncontact ACL injury. It is important to note, however, that X-ray examination has some limitations such as relatively poor imaging quality, the deviation caused by knee rotation and the condylar overlap. As opposed to this, MRI is more standardized and can consistently and accurately visualize the morphological characteristics of femoral lateral condyle. Currently, there are few studies describing the MRI-based measurement of LFCR (He and Li, 2022; Gao et al., 2023).

The primary aim of the current study was to investigate the correlation between the LFCR measured by MRI and the incidence of ACL injury. The secondary aim was to compare the LFCR measured by MRI with existing bony morphological risk factors and to assess the most predictive risk factors for an ACL rupture or rerupture.

## Materials and methods

**FIGURE 1:**
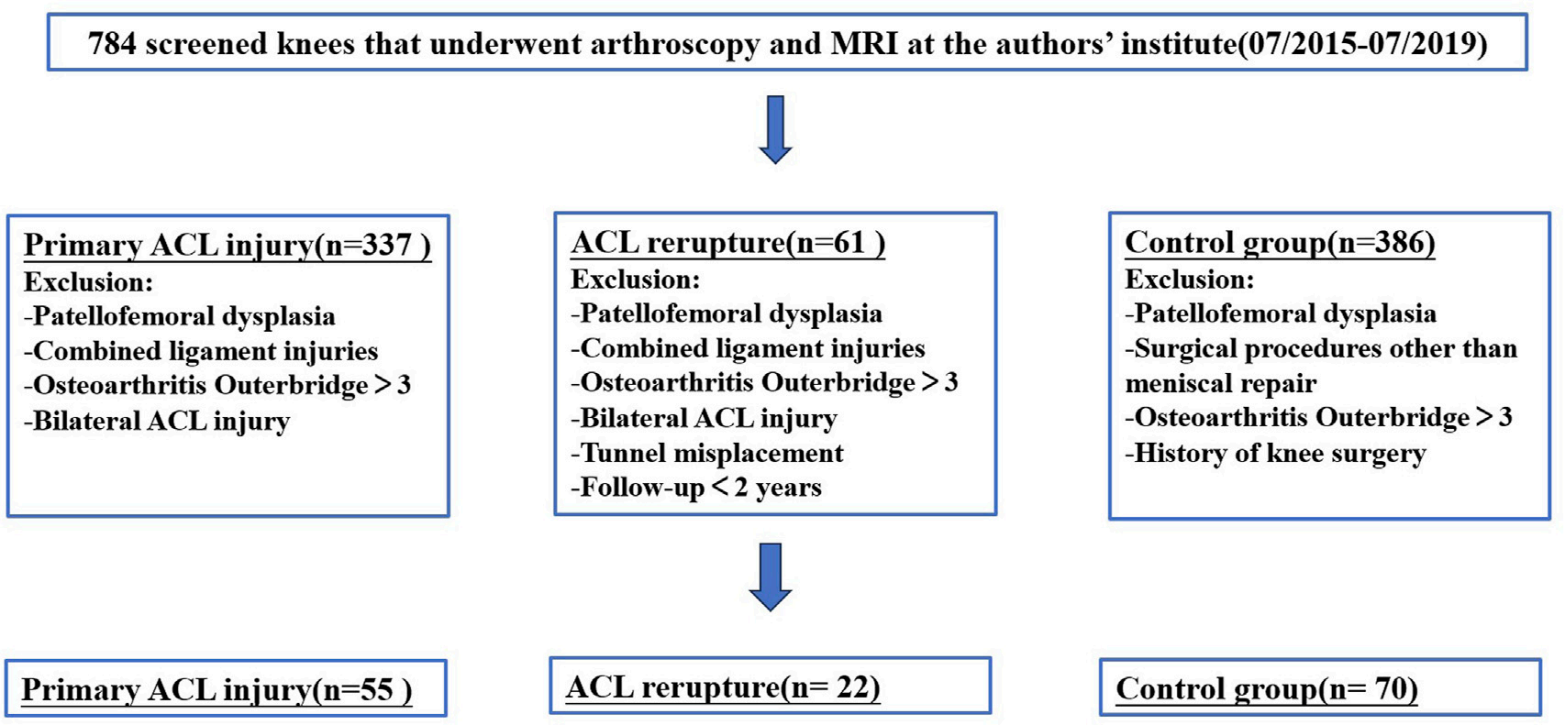
Flowchart and eligibility.

This retrospective study protocol was approved by the Medical Institutional Ethics Committee of The First People’s Hospital of Lianyungang (KY-20230914002-01). During the period July 2015 through July 2019, 784 patients who underwent knee arthroscopic surgery with the same experienced sports medicine surgeon in The First People’s Hospital of Lianyungang were enrolled in this study. The patients were divided into three groups: 1) the control group, which included patients with simple meniscus tears without ligament injury and no signs of patellofemoral dysplasia; 2) the primary noncontact ACL injury group; 3) ACL rerupture after primary ACL reconstruction (ACLR) group. Patients in different groups were matched by sex and age and then assessed in accordance with the inclusion/exclusion criteria (Figure 1). A patient could not be affiliated with multiple groups. There was a minimum follow-up period of 48 months for all patients. All the patients received MRI examination and underwent radiographic evaluation by the same imaging physician and sports medicine surgeon for evaluation of the knee injury, such as injuries of medial collateral ligament, lateral collateral ligament, posterior cruciate ligament, medial patellofemoral ligament, popliteus tendon, meniscus and cartilage.

Furthermore, it is important to point out that radiographic data of the patients who underwent ACL revision surgical procedures were reviewed to exclude ACLR failure caused by tunnel misplacement (Pinczewski et al., 2008; van Eck et al., 2012). All patients underwent MRI at 3.0 T (Siemens Medical Systems, Germany). Imaging protocols included sagittal, coronal and axial sequences. The slice thickness was 3 mm.

**FIGURE 2:**
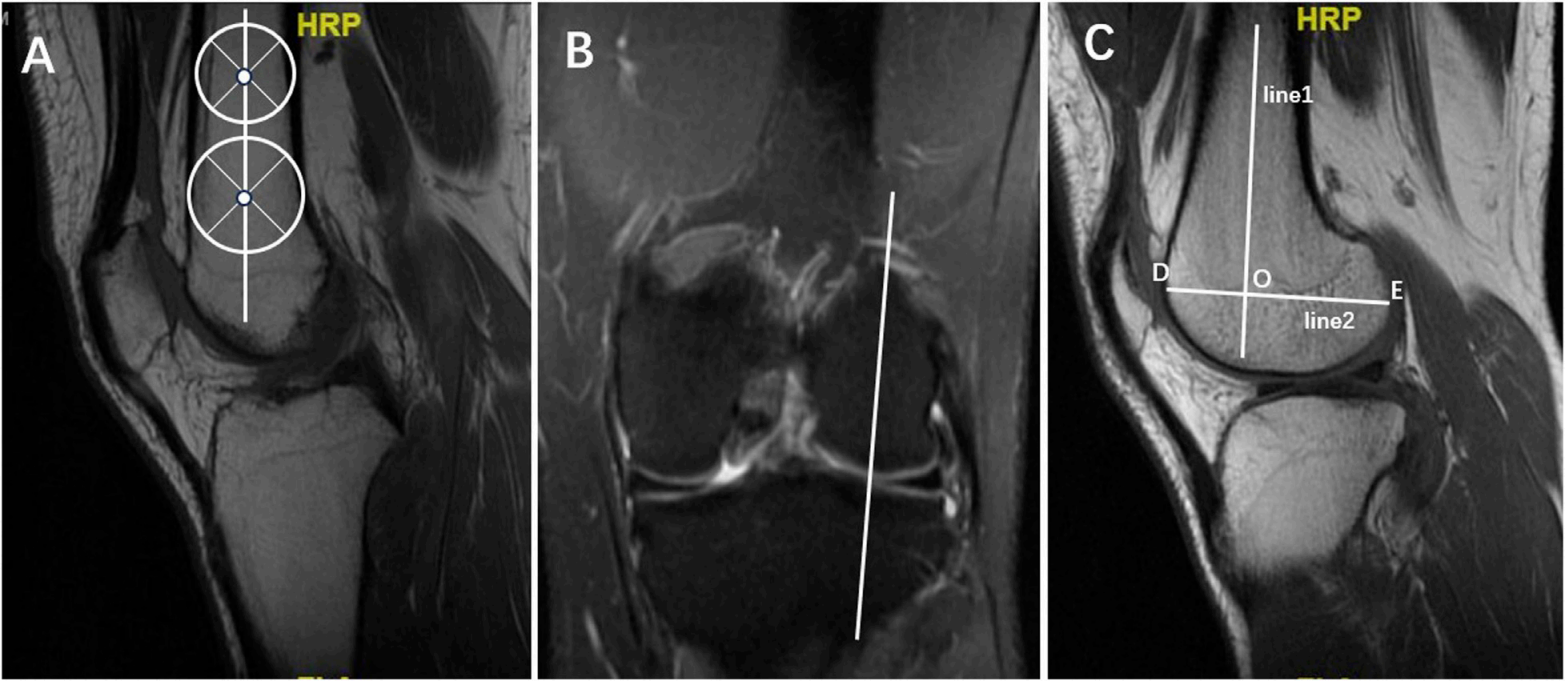
Lateral femoral condyle ratio (LFCI) measurement. **(A)** In the sagittal T1 centre of the knee MRI, two circles were drawn at the center of the distal femur. The more distant circle should be as close as possible to the trochlea of the femur. A line that passes through the centers of the two circles was thus used to determine the long axis of the distal femur (line1). **(B)** On T1 coronal sections, the midsagittal plane of the lateral femoral condyle was determined at the level of the popliteal groove. **(C)** On the corresponding sagittal slide (T1), the long axis of the distal femur (line1) was replicated and another line was drawn between the most anterior point (point D) and the last point of the lateral condyle (point E) to determine the long axis of the lateral condyle of the femur (line2). The distance from the intersection (point O) of these two lines to the last point of the lateral condyle was divided by the total length of the lateral condyle. LFCR = OE/DE.

According to the method for X-ray measurement of LFCR as Pfeiffer et al. (2018) described, LFCR was measured by MRI in this research. Through the use of coronal images (T1), the midsagittal plane of the lateral femoral condyle was determined at the level of the popliteal groove (Hodel et al., 2019; Hong et al., 2022). On the corresponding sagittal slide (T1), two circles were drawn at the center of the distal femur. The more distant circle should be as close as possible to the trochlea of the femur. A line that passes through the centers of the two circles was thus used to determine the long axis of the distal femur. Then a line was drawn between the most anterior point and the last point of the lateral condyle to determine the long axis of the lateral condyle of the femur. The distance from the intersection of these two lines to the last point of the lateral condyle was divided by the total length of the lateral condyle. This ratio was defined as the lateral femoral condyle ratio (LFCR) (Figure 2).

**FIGURE 3:**
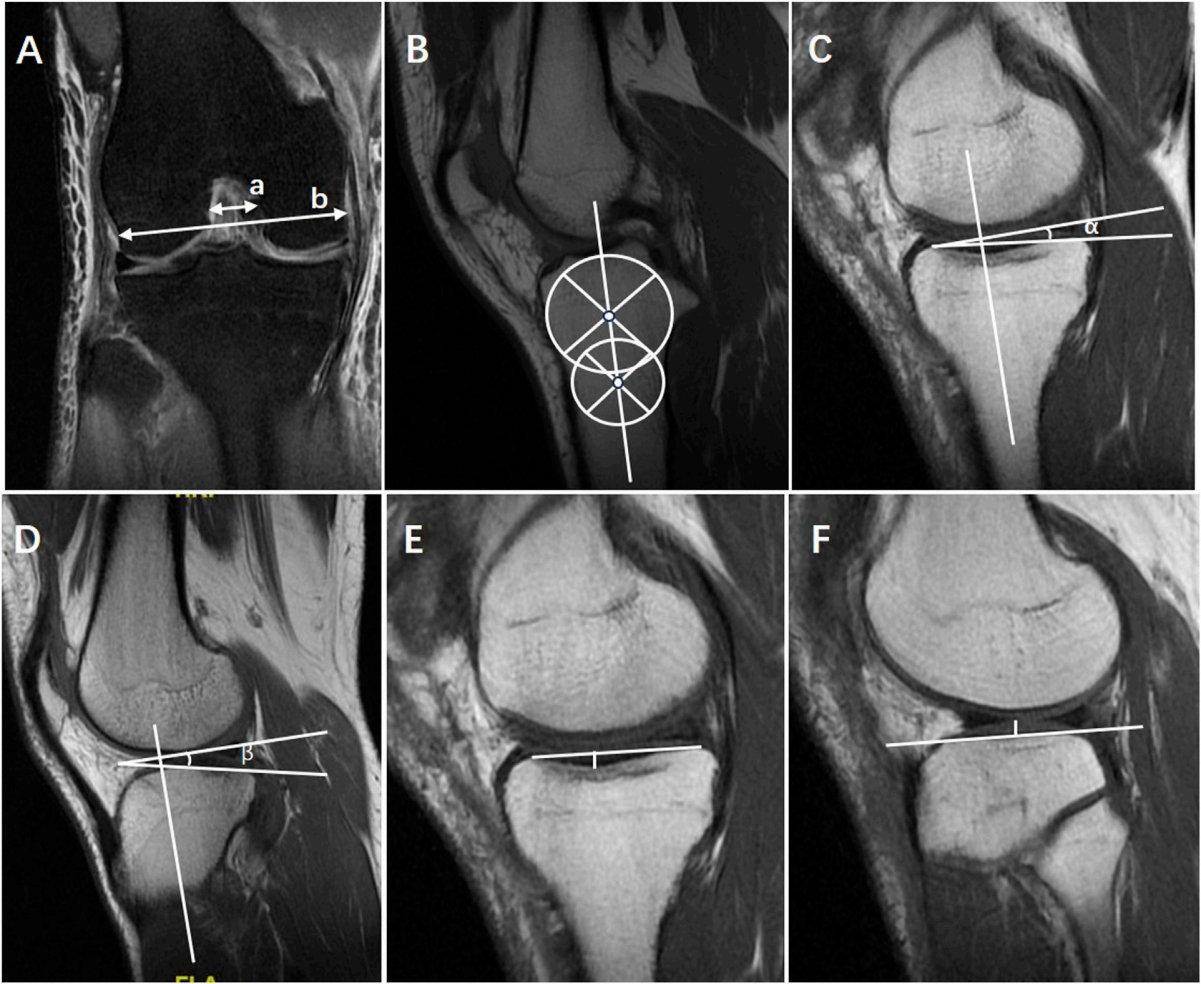
Other existing bone morphological risk factors were also measured by MRI with reference to the previous description, including **(A)** notch width index (NWI) = a/b, **(B)** the central axis of proximal tibia, **(C)** medial tibial slope (MTS) = α, on the center of the medial tibial plateau with the preserved longitudinal axis determined on the central sagittal slice, the tangent to the medial plateau was drawn to the cortex border, **(D)** lateral tibial slope (LTS) = β, on the center of the lateral tibial plateau with the preserved longitudinal axis determined on the central sagittal slice, the tangent to the lateral plateau was drawn to the cortex border, **(E)** medial tibial depth (MTD), **(F)** lateral tibial height (LTH).

In order to identify the strongest MRI predictors and construct a credible and convenient risk prediction model for ACL injury, other existing bone morphological risk factors were also performed: medial tibial slope (MTS) (Elmansori et al., 2017; Schneider et al., 2020), lateral tibial slope (LTS) (Kumar et al., 2020), medial tibial depth (MTD) (Misir et al., 2022), lateral tibial height (LTH) (Kujala et al., 1992) and notch width index (NWI) (Basukala et al., 2020; Bouras et al., 2018) (Figure 3). To evaluate the intraobserver reliability, one observer measured LFCRs in 50 patients randomly selected from the entire cohort and performed the same measurements again 2 weeks later. In order to evaluate the interobserver reliability, a second blinded observer made the measurements on the same patients independently.

Statistical analysis was conducted using the software SPSS 22.0(IBM, New York, USA) and MedCalc 12.7 (MedCalc Software, Ostende, Belgium). Statistical measures of continuous variables were presented as mean, standard deviation. A significant level was set at 0.05 (α = 0.05). The intraobserver and interobserver reliabilities were estimated with intraclass correlation coefficients (ICC). The normal distribution of the variables was assessed using the Kolmogorov-Smirnov test. As the data did not follow a normal distribution, non-parametric tests were used to analyze the bone morphological risk factors. Since there were no significant differences in the data between the ACL rerupture group and the primary ACL injury group, a pooled analysis of these two groups versus the control group was conducted. All the risk factors (LFCR, NWI, MTS, LTS, MTD, LTH) associated with *p* < 0.01 according to univariate analysis were included in the logistic regression models. Ultimately, LFCR, LTS and LTH were chosen as independent risk factors for noncontact ACL injury. The calculation encompassed not only the predictive performance of each individual risk factor but also the combined utility of LFCR, LTS, and LTH. In this study, we conducted Receiver Operating Characteristic (ROC) analysis and calculated area under the ROC curves (AUC) and 95% confidence intervals (CI) to assess the diagnostic performance. The AUC was compared with Medcalc software, and the Z-test was used to compare the predictive ability of different factors. The cutoff values were determined at maximum Youden index with optimal sensitivity and specificity.

## Results

A total of 147 patients were included in the final analysis according to the criteria for inclusion and exclusion (55 in the primary ACL injury group, 22 in the ACL rerupture group and 70 in the control group). The results of a univariable analysis revealed no significant differences between the groups in terms of age, sex, height or BMI. In this study, there was a high level of intra- and interobserver reliability for the LFCR (intraobserver ICC, 0.908; interobserver ICC, 0.945).

The LFCR in the primary ACL injury group (median, 0.628 ± 0.02; range, 0.583–0.685) and in the ACL rerupture group (0.625 ± 0.02; range, 0.599–0.680) was significantly higher compared with that in the control group (0.593 ± 0.03; range, 0.528–0.680) (*p* < 0.01). There was no statistically significant difference between the primary ACL injury group and the ACL rerupture group (*p* = 0.680). The LTS, LTH, MTS and MTD were statistically significantly higher in the primary ACL injury group and ACL rerupture group than those in the control group (Table 1). For either of the MRI measurements, there were no statistically significant differences between the primary ACL injury group and the ACL rerupture group. There were no significant differences in LFCRs based on sex among all the study groups, with a mean LFCR of 0.628 for males and 0.628 for females in the primary ACL injury group, 0.625 for males and 0.624 for females in ACL rerupture group, and 0.593 for males and 0.593 for females in the control group.

**TABLE 1:** Risk factors among groups.

	Primary ACL injury	ACL rerupture	Control group	*p*-value
LFCR	0.628 ± 0.020	0.625 ± 0.021	0.593 ± 0.030	<**0.01**
Notch Width Index	0.224 ± 0.029	0.233 ± 0.033	0.236 ± 0.025	0.13
Medial Tibial Slope	6.13 ± 3.09	6.89 ± 2.72	5.345 ± 2.930	0.029
Lateral Tibial Slope	8.206 ± 2.956	8.5 ± 3.42	6.33 ± 3.2	<**0.01**
Medial Tibial Depth	2.374 ± 0.922	2.5 ± 0.89	2.035 ± 0.904	0.028
Lateral Tibial Height	3.863 ± 0.567	3.743 ± 0.64	3.088 ± 0.663	<**0.01**

Note: Values are presented as mean, standard deviation. A pooled analysis of the two groups (the primary ACL injury group and the ACL rerupture group) versus the control group was conducted and *p* values < 0.05 are marked bold.

**FIGURE 4:**
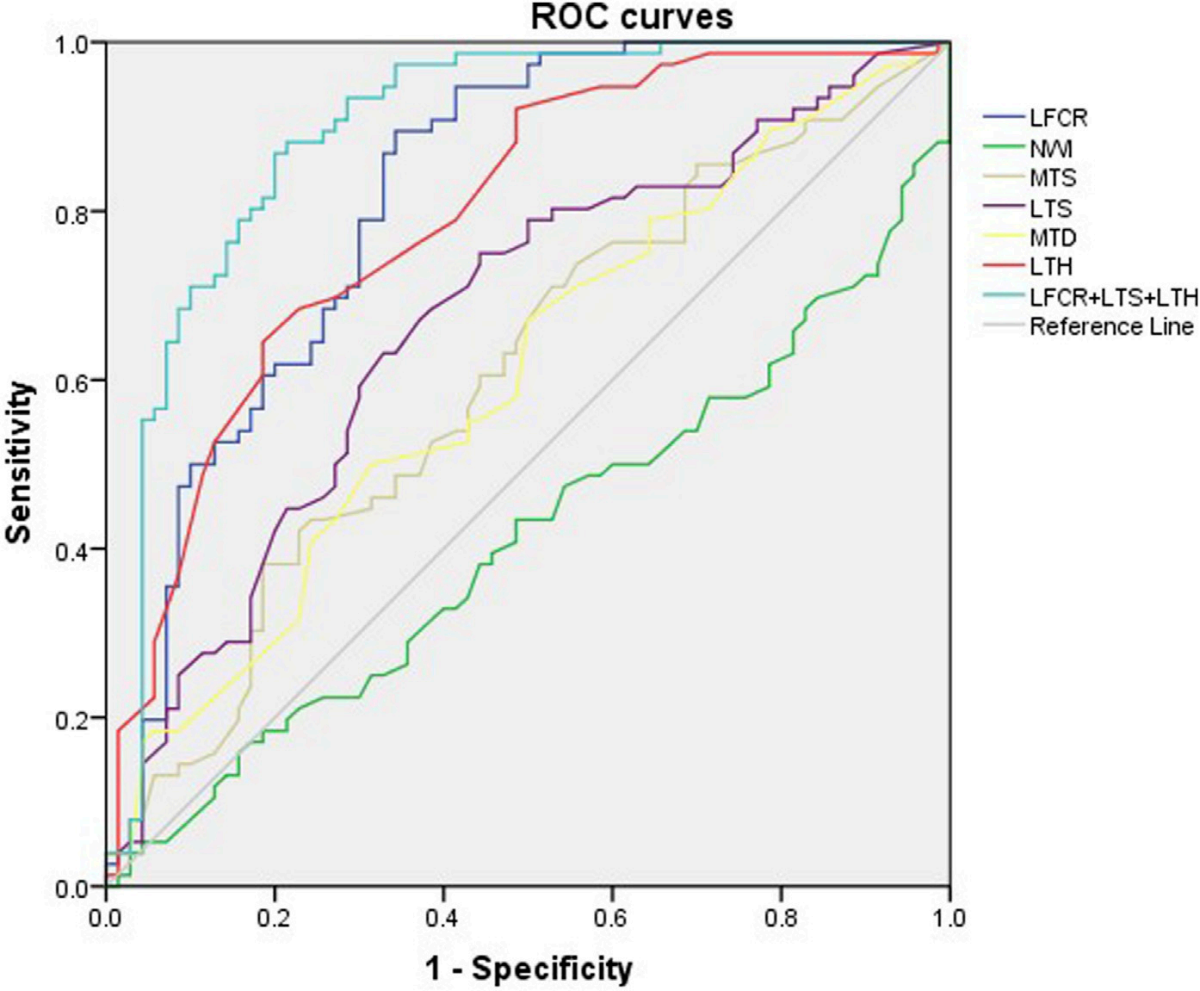
Diagnostic performance of the risk factors (LFCR, NWI, MTS, LTS, MTD, LTH, LFCR+LTS+LTH) was evaluated using receiver operating characteristic curves (AROC) and the reference line was AUC = 0.5. AUC, area under the curve; LFCR, lateral femoral condyle ratio; NWI, notch width index; MTS, medial tibial slope; LTS, lateral tibial slope; MTD, medial tibial depth; LTH, lateral tibial height.

Logistic regression analysis was used to screen out the risk factors for ACL injury, including LFCR (*p* < 0.01), MTS (*p* = 0.029), LTS (*p* < 0.01),MTD (*p* = 0.028), LTH (*p* < 0.01). LFCR, LTS and LTH were screened as independent risk factors for ACL injury. Further analysis of the ROC curves of all the risk factors showed statistical significance (Figure 4). The highest AUC was reported for LFCR (0.818; 95% CI, 0.748–0.878) to predict ACL injury or rerupture, and the sensitivity and specificity were 89.61% and 65.71%. A cutoff value of 0.602 (Youden index = 0.553) was related to a higher risk of ACL injury or rerupture. Among the remaining factors, the AUC of LTS (0.672; 95% CI, 0.583–0.741) (*p* < 0.001) and LTH (0.794; 95% CI, 0.720–0.857) (*p* < 0.001) were significantly larger than that of 0.5. The combination of LFCI, LTS and LTH by logistic regression yielded a higher AUC of 0.896 (95% CI, 0.890–0.950) and a higher specificity of 80% compared to those of LFCI alone (Table 2).

**TABLE 2:** Diagnostic performance.

	LFCR^a^	Medial Tibial Slope	Lateral Tibial Slope	Medial Tibial Depth	Lateral Tibial Height	Combination^b^
Sensitivity%	90	38	74	49	65	87
Specificity%	66	81	56	69	81	80
AUC	0.818	0.605	0.672	0.605	0.794	0.896
AUC: 95% CI	0.748–0.878	0.514–0.678	0.583–0.741	0.520–0.684	0.720–0.857	0.890–0.950
*p*-value						
AUC = 0.5^c^	<0.001	0.029	0.0002	0.0257	<0.0001	<0.0001
LFCR^d^	——	0.0004	0.0077	0.0001	0.5842	0.0044
Cut-off value	0.602	7.6	7	2.5	3.6	0.780
Youden index	0.553	0.191	0.297	0.179	0.464	0.670

^a^ AUC, area under the ROC curve; LFCR, lateral femoral condyle ratio.

^b^ Combination = LFCR+ lateral tibial slope+ lateral tibial height.

^c^ *p* value of each AUC tested against 0.5; binomial Z-test.

^d^ *p* value of each AUC tested against the AUC for the LFCR; binomial Z-test.

## Discussion

This study indicated that an increased lateral femoral condyle ratio (LFCR) measured by MRI was significantly associated with an increased prevalence rate of ACL injury or rerupture. The cutoff value of 0.602 for LFCR, with a sensitivity of ≥90% and a specificity of ≥66% for identifying ACL injury might help clinicians predict the potential risk of ACL injury and rerupture. The combination of the three most predictive factors (LFCR> 0.602, LTS>7° and LTH >3.6 mm) further enhanced the diagnostic performance for ACL injury, with an AUC of 0.896, a sensitivity of 87%, and a specificity of 80%. LFCR measured by MRI was proved to be an accurate, reliable, highly sensitive and reproducible indication for ACL injury.

A significant relationship has been found between the shape of the distal femur and knee joint movement (Bayer et al., 2020; Hodel et al., 2022; Pfeiffer et al., 2019). A larger LFCR means a flatter front of the lateral condyle of the femur and the depth of the posterior condyle is larger. As the limb changed position from safe to provocative to exaggerated provocative, the point of contact moved further anteriorly from the posterior tibial point. During the exaggerated provocative position, the lateral femoral condyle contacted the tibial plateau on its flat anterior surface rather than its rounder posterior surface. This might result in a greater likelihood for sliding instead of rolling, which could strain or tear the ACL with a compressive force (Boden et al., 2009).

There was no statistically significant difference in LFCRs between patients in the primary ACL injury group and in the ACL rerupture group. This might be attributed to three reasons. First, although postoperative CT excluded the possibility of nonanatomic tunnel placement, the risk for an ACL rerupture could be biased by a suboptimal tunnel placement (Hodel et al., 2019; Pinczewski et al., 2008). During ACL reconstruction in this study, as soon as the knee was hyperflexed, the femoral tunnel was drilled through the ACL femoral footprint. The entry of femoral tunnel was either at 2 or 10 o’clock position for left and right knee respectively. In the center of the ACL anatomical tibial footprint, located at the posterior border of the anterior horn of the lateral meniscus, the tibial tunnel was drilled with ACL tibial tunnel guide. Adouni et al. (2023) used a global sensitivity analysis based on variance decomposition to investigate the contribution of the surgical parameters to the uncertainty in response to the joint after ACL reconstruction surgery. Their results indicated that the joint contact center and area were affected mainly by the angle of fixation and the tunnel placements. It was important for surgeons to carefully construct anatomical tunnels with right dimension during ACL reconstruction since the tunnel dimension and the angle can also affect the rate of re-rupture (Vermeijden et al., 2020; Byrne et al., 2022; Snaebjornsson et al., 2017). Second, the insufficient follow-up time may be another disadvantage. We will further expand the sample size and prolong the duration of follow-up in future work in order to obtain more data to explore this correlation between the LFCR and the subsequent risk for ACL rerupture. Third, the effect of collagen diameter on ACL injury and rerupture needs further study. Researchers have determined the collagen fibril diameter distributions in healthy and injured ACL tissue of rat or sheep and created polycaprolactone (PCL) constructs to mimic the distributions of collagen fibrils in healthy and injured tissues successfully (Adeoye et al., 2022; Smatov et al., 2023). Moreover, Pfeiffer et al. (2018) also found that a profoundly increased LFCR could increase the risk for contralateral ACL injury compared with no ACL injury or primary ACL injury. In light of the results here, we anticipate a greater focus on this area of research in the future.

In addition, the risk factors analysis revealed a no significant difference in LFCR between men and women, which is contradictory to some current research results (Pfeiffer et al., 2018; Pinskerova et al., 2014). Pfeiffer et al. (2018) concluded that the LFCR measured by X-ray was significantly higher in female patients (64.6%) than in male patients (63.1%). This may explain why female athletes were at greater risk of ACL injury than male athletes (Montalvo et al., 2019). However, other studies denied this significance (He and Li, 2022; Hodel et al., 2019). This discrepancy may be due to the limited number of studies and sample size. Therefore, future studies are needed to verify these results.

Previous studies pointed out that as a result of excessive LTS and LTH, the tibia will move forward, the knee joint will become unstable, and the ACL will become tensed, increasing the risk of ACL injury (Chen et al., 2022; Kujala et al., 1992). Tat et al. (2021) mathematically modeled the posterior tibial plateau geometry in patients and found that patients with ACL injuries had a significantly greater posterolateral plateau slope than that of matched controls. The steeper drop off may result in higher anterior translation forces, coupled with internal rotation torques on the knee, which could increase ACL axial strain. Shi et al. (2024) performed a retrospective analysis on 52 patients undergoing primary or revision ACL reconstruction. It was found that compared to patients with intact ACLR, those who sustained nontraumatic ACL reconstruction failure showed significantly increased LTS, MTS and deceased NWI. The related biomechanical mechanism remains a hotspot of current research.

The results of this study suggest a higher risk of the prevalence of ACL injury or rerupture in patients with an increased LFCR, LTS and LTH. This matches the results previously reported and confirms our previous hypothesis that the tibial and femoral bony morphology of the lateral compartment together impact the rotatory stability as well as the anterior-posterior stability of knee. The combination of the three risk factors was most predictive for ACL injury. The prediction model may help clinicians to identify patients at high risk for ACL injury or rerupture who could benefit from a more precise and personalized risk assessment, surgical indication decision, additional extra-capsular reconstruction, adjustment of graft selection, modification of surgical techniques (Choi et al., 2023; Pfeiffer, 2023), prediction of postoperative outcomes and individualized precision rehabilitation therapy (Fu et al., 2021). However, how the three risk factors studied here could influence the biomechanical responses of the knee joint remains worthy of further study. Adouni et al. (2020) presented a novel iterative method for computing the *in situ* strain at reference configuration. This framework used an *in situ* strain gradient approach (deformed reference configuration) as well as a detailed finite element (FE) model of the knee joint. Then the effect of the risk factors on the biomechanical responses of the joint could be well investigated during the passive extension and flexion motions, the axial compression and the coupled internal–external rotations.

Numerous studies have proposed various methods for describing the bone morphological characteristics of the lateral compartment and a lot of imaging measurements have been screened out for assessing the risk of ACL injury, such as LFCI, NWI, MTS, LTS, MTD and LTH. Pfeiffer et al. (2018) measured LFCR by X-ray and prove it to be a reliable and independent risk factor for ACL injuries. The advantage of this method is that it is convenient, fast and cheap. However, a sizeable number of patients had to be excluded prior to analysis because of malrotated radiographs in that study. Voleti et al. (2014) believe that as a result of both articular cartilage thickness and the anatomic differences between medial and lateral condyles, Plain radiographs underestimate the posterior condylar depth measurements as well as the asymmetry of the medial and lateral posterior condylar offset compared with MRI. In order to avoid this as much as possible, we proposed a new method of measurement using MRI. MRI exhibits minimal radiation and possesses the capability to assess numerous imaging measurements which are connected with the bone morphological characteristics of the lateral compartment, albeit at a higher cost and with increased time requirements compared to X-ray techniques. In assessing the lateral condyle of the femur, LFCR measured by MRI is independent of rotation and tilt of the femur, which demonstrates high repeatability and reliability.

## Conclusion

This research concludes that an increased LFCR measured by MRI is significantly associated with increased incidence of ACL injury or rerupture, and the LFCR, LTS and LTH are the most predictive risk factors for ACL injury. This may ultimately help clinicians identify those individuals at greatest risk of ACL injury, who may greatly benefit from prevention counseling, surgical or nonsurgical intervention and personalized postoperative rehabilitation protocol.

## Data Availability

The original contributions presented in the study are included in the article/supplementary material, further inquiries can be directed to the corresponding author.
